# Occurrence and Abundance of Antibiotics and Resistance Genes in Rivers, Canal and near Drug Formulation Facilities – A Study in Pakistan

**DOI:** 10.1371/journal.pone.0062712

**Published:** 2013-06-28

**Authors:** Ghazanfar Ali Khan, Björn Berglund, Kashif Maqbool Khan, Per-Eric Lindgren, Jerker Fick

**Affiliations:** 1 Department of Chemistry, Umeå University, Umeå, Sweden; 2 Linköping University, Division of Medical Microbiology, Department of Clinical and Experimental Medicine, Linköping, Sweden; 3 College of Pharmacy, University of Punjab, Lahore, Pakistan; 4 Medical Diagnostics, County Hospital Ryhov, Jönköping, Sweden; Oak Ridge National Laboratory, United States of America

## Abstract

Antibiotic resistance (AR) is a global phenomenon that has severe epidemiological ramifications world-wide. It has been suggested that antibiotics that have been discharged into the natural aquatic environments after usage or manufacture can promote the occurrence of antibiotic resistance genes (ARG). These environmental ARGs could serve as a reservoir and be horizontally transferred to human-associated bacteria and thus contribute to AR proliferation. The aim of this study was to investigate the anthropogenic load of antibiotics in Northern Pakistan and study the occurrence of ARGs in selected samples from this region. 19 sampling sites were selected; including six rivers, one dam, one canal, one sewage drain and four drug formulation facilities. Our results show that five of the rivers have antibiotic levels comparable to surface water measurements in unpolluted sites in Europe and the US. However, high levels of antibiotics could be detected in the downstream river in close vicinity of the 10 million city Lahore, 1100, 1700 and 2700 ng L^−1^ for oxytetracycline, trimethoprim, and sulfamethoxazole respectively. Highest detected levels were at one of the drug formulation facilities, with the measured levels of 1100, 4100, 6200, 7300, 8000, 27000, 28000 and 49000 ng L^−1^ of erythromycin, lincomycin, ciprofloxacin, ofloxacin, levofloxacin, oxytetracycline, trimethoprim and sulfamethoxazole respectively. ARGs were also detected at the sites and the highest levels of ARGs detected, *sulI* and *dfrA1*, were directly associated with the antibiotics detected at the highest concentrations, sulfamethoxazole and trimethoprim. Highest levels of both antibiotics and ARGs were seen at a drug formulation facility, within an industrial estate with a low number of local residents and no hospitals in the vicinity, which indicates that the levels of ARGs at this site were associated with the environmental levels of antibiotics.

## Introduction

During the last two decades, occurrence of antibiotics into water bodies and subsequent development of resistance in microorganisms have come into scientific and public focus as an issue of potential concern [1]–[3]. The growing increase in resistance, as established today, is considered to be closely linked with the widespread misuse and overuse of antibiotics in humans, animals and agriculture [4]. There are several sources that can contribute significantly to the environmental burden of antibiotics such as hospital waste, waste water treatment plants (WWTPs), sewage treatment plants (STPs), inappropriate disposal of unused drugs, veterinary and agriculture (animal husbandry and aquaculture) use [5]–[14]. Elevated levels of antibiotics in the environment could enhance the development of antibiotic resistance genes (ARGs) due to natural selection and also help establish the environment as a reservoir for further propagation and proliferation of ARGs to pathogens via water and food webs.

Discharge from basic drug manufacturing facilities (BDMF) and drug formulation facilities (DFF) have been considered of minor importance but recent studies have shown that they can be substantial point sources with levels much higher than any other route. On the basis of contribution from such sources, one may categorize them as levels up to mg L^−1^ [15]–[18], >100 µg L^−1^ [19]–[21] and <1 µg L^−1^ [22]–[26]. The highest levels ever reported from BDMF is 31 mg L^−1^ for ciprofloxacin (fluoroquinolone) from WWTP effluent receiving polluted water from about 90 bulk drug manufacturers in Patancheru near Hyderabad, India [15]. In addition to this, a metagenomic study of the waste water effluent from Patancheru showed presence of antibiotic resistance genes belonging to several classes of antibiotics, including sulfonamides, fluoroquinolones and aminoglycosides along with class 1 and 2 integrons [27].

Horizontal gene transfers (HGTs) are the main mechanisms through which ARGs are exchanged among bacteria of diverse origin including environmental, non-pathogenic, human pathogenic, gram- positive and negative via mobile DNA elements such as plasmids and transposons – either with or without mobile integrons [28], [29]. Mobile integrons have the ability to recognize and acquire one or more gene cassettes containing ARGs [30] while chromosomal integrons (superintegrons) are associated with chromosomes carrying large number of gene cassettes [31]. The involvement of class1 integrons in the spread of ARGs both in environment and human pathogens is viewed as a serious challenge to clinical therapy [12], [32]. Consequently increasing incidences of appearance of resistances in some clinically important pathogens such as methicillin resistance *Staphylococcus aureus* (MRSA), vancomycin resistant enterococci (VRE), *Clostridium difficile*, *Klebsiella pneumonia*e and *Acinetobacter baumannii* are the clear manifestation of growing problem. In addition to this, metals contribution in the proliferation of ARGs by co-selection and cross-resistance has also been reported [33], [34].

ARGs are not only detected in sewage, surface water, oceans, sediments and soils [35] but also have been found in wide variety of environments such as remote Alaskan soil [36], deep terrestrial subsurface [37], the deep Greenland ice core [38]and the waters of the Antarctic Ocean [39]. The contribution of BDMF to antibiotic resistance development has been reported by Li et al. showing ARGs encoding for ß-lactamases and class 1 integrons from penicillin production WWTP effluent [40] and tetracycline ARGs and class 1 integrons from oxytetracycline production WWTP [41]. Similarly contribution from drug production facilities has also been reported in Denmark and Cuba [42], [43].

The purpose of the study was to investigate the occurrence and abundance of antibiotics in Pakistan's rivers and the effluent of DFFs and to further evaluate the impact of a large size city (Lahore) on the abundance of ARGs and the concentration of antibiotics in the environment.

## Materials and Methods

### Chemicals and standard

Sulfadiazine (SDZ), Lincomycin (LIN), Trimethoprim (TRI), Enoxacin (ENX), Oxytetracyclin (OXY), Ofloxacin (OFL), Levofloxacin (LEV), Norfloxacin (NOR), Pefloxacin (PEF), Ciprofloxacin (CIP), Cefotaxime (CEF), Lomefloxacin LOM), Tetracycline (TET), Enrofloxacin (ENR), Azithromycin (AZI), Clindamycin (CLI), Sulfamethoxazole (SULM), Doxycycline (DOX), Erythromycin (ERY), Nalidixic acid (NAL), Clarithromycin (CLA) and Roxithromycin (ROX) were obtained from Sigma Aldrich (Steinheim, Germany). All drugs were classified as HPLC grade (>98%). Formic acid (puriss pa) and sulphuric acid were acquired from Fluka (Steinheim, Germany) and Merck (Dramstadt, Germany) respectively. Methanol, acetonitrile and water (Lichrosolv, Hypergrade) were obtained from Merck Darmstadt, Germany). Purified water (resistivity: 18.2 MΩcm) was prepared by passing water through an ELGA MAXIMA HPLC ultra-pure water system (ELGA, High Wycombe Bucks, England) equipped with a UV radiation source. The internal standards used were obtained from Cambridge Isotope Laboratories (Andover, MA, USA): ^13^C_2_-Trimethoprim (^13^C_2_-TRI) (99%), ^13^C_3_15N-Ciprofloxacin (^13^C_3_-CIP) (99%), ^13^C_6_-Sulphamethoxazole (^13^C_6_-SULM) and ^13^C_2_-Erythromycin (^13^C_2_-ERY).

### Background and sampling sites

**Figure 1:**
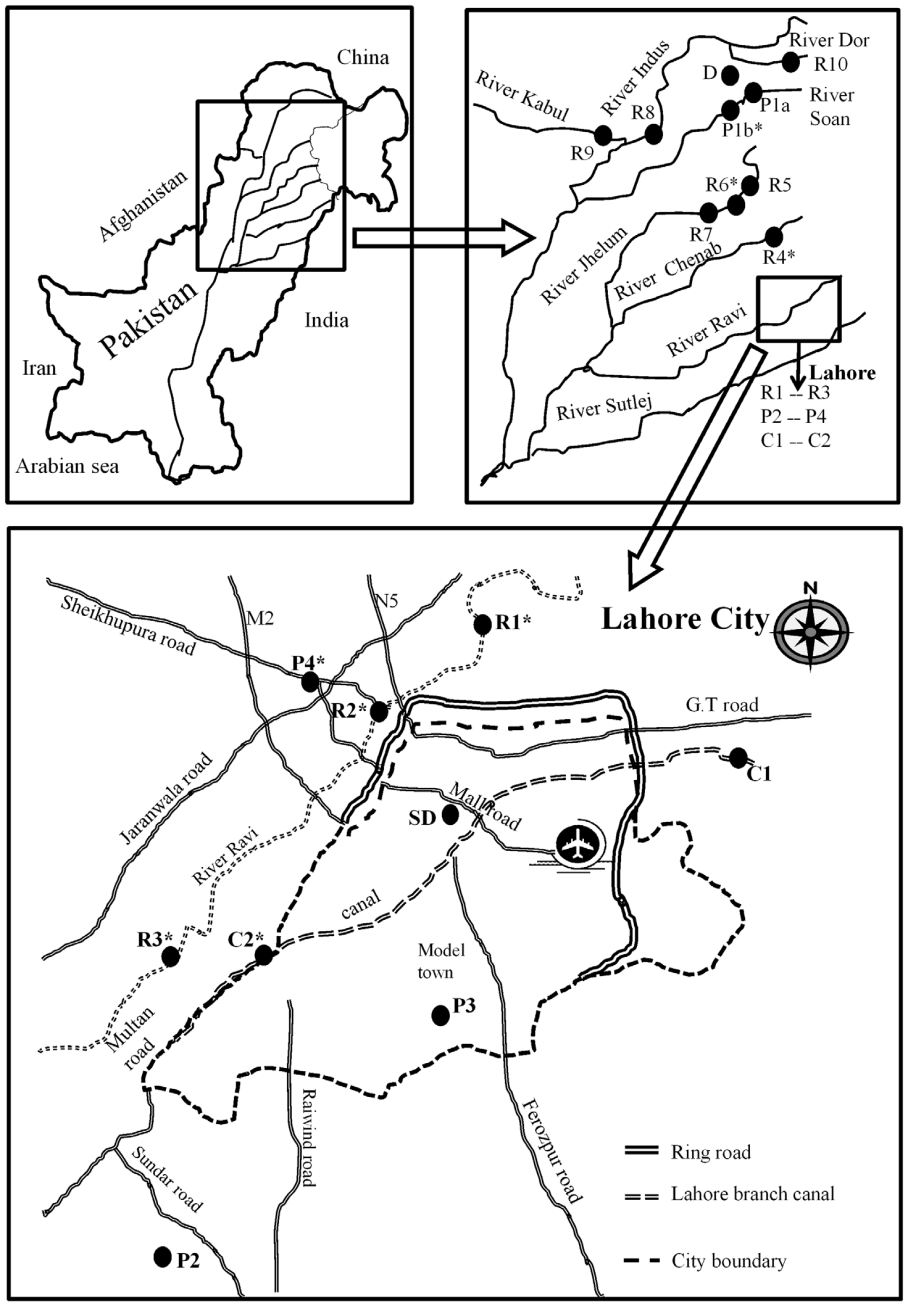
Map of the region with sampling sites indicated. R1–R10 are rivers, D is the Rawal dam, C1–C2 is the Lahore branch canal, SD is the sewage drain nearby hospitals and P1–P4 are the drug formulation facilities; *: sites where sediment samples are also taken along with water samples.

Although Pakistan has made progress towards semi-industrialized economy yet agriculture is the mainstay providing employment to around 45% of the population. Consequently rivers and in particular the River Indus, provides key water resources and is considered the backbone of agriculture. The River Indus is approximately 2900 km long and all other major rivers such as the Ravi, the Jhelum and the Chenab join it from north-eastern side (Figure 1).

Altogether nineteen sampling sites were selected on six rivers, one dam, one canal, one sewage drain and four DFFs (Figure 1). The rivers included the River Ravi, the Jhelum, the Chenab, the Indus, the Kabul and the Dor. On the River Ravi and the River Jhelum, three sites were selected; R1 is approximately 6 Km upstream Lahore city, R2 is situated close to the city center and R3 is 19 Km downstream. Sampling site R4 is near the bridge on the River Chenab on the Grand Trunk (G.T) Road, close to the city Gujrat. Sampling site R5 is 9.5 Km upstream the Jhelum city, R6 is in the vicinity of the Jhelum city center and R7 is 10 Km downstream. R8, R9 and R10 are on the River Indus, the Kabul and the Dor respectively. D is a site at the Rawal Dam in Islamabad, SD is a site in Lahore at a sewage drain located nearby Shadman market receiving household and hospitals effluent from three major hospitals namely Government Institute for Mental Health, Services Hospital and Punjab Institute of Cardiology. Two sampling sites were located on the Lahore Branch Canal, which passes through the city; one at the entrance, C1 (Jhalomor), and the other at a distance of 30 km outside the city, C2 (13 Km Multan Road). Lahore is densely populated city with unofficial population figures estimated at around 10 million. One DFF site in Islamabad and three in and around the Lahore city were selected. P1a is located south of Islamabad on the River Soan, upstream the Kahuta Industrial Estate (KIE) and P1b is located downstream the KIE on the same river. P2 is located at Sundar Industrial Estate approximately 25 Km to the Southwest of Lahore. P3 is located at Quaid-e-Azam Industrial Estate (Kotlakhpat) in the South of Lahore and P4, at Shahdara Industrial Estate located Northwest of Lahore. Sediment samples were taken at sampling sites R1–R4, R6, C1, P1b and P4. Sampling sites coordinates and sampling dates are presented in the Table S1.

### Sample collection and preparation

At each sampling site, surface water grab subsamples were collected at 3–5 points, across equally distant width of the water body and at a depth of approximately 0.5 m. In order to ensure uniformity, equal quantities of each individual subsamples were pooled together to make one large sample before finally filled into 120 ml amber coloured polyethylene plastic bottles for storage and transportation in an ice box. Similarly grab water samples were collected near DFFs. The samples were freezed before shipment and stored at −20°C until analysis. Similarly, 5 subsamples of sediments were pooled before storage in clean plastic tubes. Samples of water were taken once at R4, R8, R9, R10 and D, twice at C1 and C2 and thrice at the rest of the sites, whereas sediment samples were taken once.

10 ml of thawed water samples were filtrated using syringe driven filter units 0.22 µm (Millipore) and fortified by 0.1 ng ml^−1^ of each of the internal standards. 50 ng of each internal and surrogate standard were added to the sediment samples (0.1 g, dry weight) used for extraction. Sediment extraction was sequentially performed, first using 1.5 ml ethyl acetate and methanol (1∶1 mixture) followed by 1.5 ml methanol and water (7∶3 mixture) with 5% triethylamine. Samples were homogenized for four minutes, at 42000 oscillations per minute, using a Mini Beadbeater (Biospec. Bartlesville, USA) with zirconium beads and then centrifuged at 16000 g for 10 min. This protocol was done for both eluent mixtures and the supernatants were combined, evaporated to 20 µL and reconstituted in 1 ml water and acetonitrile (95∶5 mixture) with 0.1% formic acid.

### LC-MS and quantification

Antibiotics included in this study (Table S2) were selected based on their registered usage in Pakistan and the method was based on the previous protocol Khan et al. 2012 [44]. Identification of suitable precursor ions for new analytes was done by recording a full scan in both positive [M+H^+^] and negative [M+H^−^] at MRM (multiple reaction monitoring) modes by direct infusion of 1 µ L^−1^ standard solution at a flow rate of 250 µL min^−1^ into ion source followed by full product ion scans of selected precursor ions. For quantitative optimization the Xcalibur software package (rev. 2.0 SP2, Thermo Fischer Scientific) was used for determining the product ions and their corresponding declustering potentials, collision energies and exit potentials. The product with highest intensity was taken as the quantitative transition and the second highest as the confirmation transition.

### Quantification and quality assurance

Quantification was achieved using internal standard (IS) calibration; target analytes were identified on the basis of their similarity to the IS in terms of structure, retention time, and molecular weight. Several calibration standards covering all concentrations range were measured before, in the middle and at the end of sample sequences. The maximum difference between results at quantification and qualification mass transition was set to 30% as criterion for positive identification.

### DNA extraction

DNA was extracted from 150 mg sediment using FastDNA SPIN Kit for Soil and the FastPrep Instrument (MP Biomedicals, Santa Ana, CA) according to the manufacturer's protocol. Extracted DNA was stored in −20°C before analysis. The total amount of extracted DNA were quantified using Quant-iT™ PicoGreen dsDNA reagent (Invitrogen, Carlsbad, CA) to make sure that the DNA yield corresponded linearly to the starting amount of sediment.

### Quantification of antibiotic resistance genes using real-time PCR

Quantitative real-time PCR was employed on the extracted DNA to quantify five different antibiotic resistance genes; tetracycline resistance genes *tetA* and *tetB*, sulphonamide resistance gene *sulI*, trimethoprim resistance gene *dfrA1* and macrolide-, lincosamide- and streptogramin B resistance gene *ermB*. Additionally, the gene coding for integrase on class 1 integrons, *intI1*, was quantified. All measured gene quantities were normalized against the 16S rRNA gene content in the respective sample, which was also measured using real-time PCR. Primers and cycling conditions used are presented in Table S4.

Real-time PCR quantification was performed using serial dilutions of amplicon-carrying pUC57 plasmids (Genscript, Piscataway, NJ) ranging from 6 to 10^6^ gene copies, as standard. Plasmid concentrations were measured using NanoDrop 2000 (Thermo Fisher Scientific, Waltham, MA) and Quant-iT™ PicoGreen dsDNA reagent. Plasmids were linearized with FastDigest® *Eco*RI (Fermentas, Vilnius, Lithuania) before use.

All real-time PCRs were performed on a CFX96™ Real-Time PCR Detection System (Bio-Rad Laboratories, Hercules, CA). 5 µl DNA was used in the reaction mixture, which consisted of primers and master mix, to a total of 20 µl. Maxima® SYBR Green qPCR Master Mix (Fermentas) was used for the reaction mixtures of the assays using the SYBR Green detection format (16S rRNA gene, *sulI*, *dfrA1*, *ermB* and *intI1*). For the Light-Upon-eXtension (LUX) assays (*tetA* and *tetB*), Platinum® Quantitative PCR SuperMix-UDG (Invitrogen) was used. All primers were purchased from Sigma-Aldrich, except LUX primers, which were purchased from Invitrogen.

### Statistical analysis

Friedman tests and Dunn's multiple comparison tests performed post hoc were used to determine differences in levels of antibiotics and ARGs between the different sampling sites. Antibiotics were tested separately from ARGs and the significance level was set to p<0.001 due to the large number of comparisons. Differences between sampling sites R1, R2 and R3 were of particular interest as they were taken upstream, inside and downstream of Lahore and would indicate any influences of the city on the contamination levels. Therefore these sites were additionally tested separately with significance levels p<0.05. All statistical analyses were performed in Prism 5 for Windows v5.00.

### Ethical Statement

No specific permits were required for the described field studies. There are no regulations restricting taking water from rivers, a canal, a dam or near drug formulation facilities. The sampling sites are not privately owned. The field study does not involve any endangered or protected species and only water and sediment samples were taken from the sites.

## Results

### Optimization and QA/QC

The quantitative and confirmation transitions monitored for all analytes are listed in Table S2. As all the compounds exhibited highest intensities in positive ion mode, HESI (+) was used throughout the analysis. The analytical method used was stable throughout the study, all retention times were within 2% of the standards, linearity for all standard curves were >0.99 and no memory effects or cross talk could be detected.

### Determination of antibiotics in rivers, canal, dam and sewage drain

Results of measured levels of antibiotics in ng L^−1^ for rivers, the canal, the dam and the sewage drain close to hospitals are presented in Table 1. It should be noted that the relative standard deviation (RSD) presented in Table 1, reflects the variance in usage since the samples were taken with a gap of approximately once a week. All antibiotics included in the study were detected, however most antibiotics were only measured at levels well below 100 ng L^−1^. Sampling sites R1, R4–R10 could be classified as low level sites (with sites R5–R10 ranking significantly lower in overall antibiotic levels than R3, P3, P4, P1b and SD with p<0.001) with exception for SULM that were detected in the range of <limit of quantification (LOQ) – 170 ng L^−1^. In the river sampling sites, the measured levels ranged from <LOQ – 2700 ng L^−1^ and the highest levels were seen at R3, i.e. downstream Lahore city in the River Ravi. Our results show an increasing trend in measured levels from R1 to R3 (p<0.01), i.e. before and after Lahore city, which indicate the impact of the municipal sewage waste from the 10 million city Lahore. Antibiotic levels in R3 were overall very high as compared to the other sites as well, ranking higher than sites R5–R10 and D (p<0.001). Levels exceeded 1 µg L^−1^ on three occasions at R3; 1100, 1700 and 2700 ng L^−1^ for OXY, TRI and SULM respectively. Fluoroquinolones were not detected at high levels, the range for the nine fluoroquinolones included in the study was <LOQ – 120 ng L^−1^, and only on two occasions did measured levels exceed 100 ng L^−1^ (R3). Lowest levels of antibiotics were measured at R8, R9 and R10, which were considered to be the most pristine sites. Low levels were also detected at the Rawal Dam (D) (overall antibiotic levels significantly lower than in sites R3, P3, P4, P1b and SD with p<0.001) with measured levels that ranged from <LOQ – 16 ng L^−1^, with the highest concentration of DOX. At C1, the inlet of the Lahore Branch Canal, the range of measured levels was <LOQ – 46 ng L^−1^ and an increasing trend in measured concentrations was observed from C1 to C2 (although not statistically significant), where the range was <LOQ – 300 ng L^−1^. The range of average measured levels at SD, a sewage drain close to Shadman market receiving household and hospitals effluent from three hospitals, was <LOQ – 4600 ng L^−1^ with highest detected levels for SULM. Levels of four antibiotics exceeded 1 µg L^−1^ i.e. 1100, 2200, 3200 and 4600 ng L^−1^ for LIN, TRI, OXY and SULM respectively. Moreover levels also exceeded 100 ng L^−1^ for OFL, LEV, CIP, DOX, ERY and NAL. Antibiotic levels at SD ranked significantly higher than at sites R5–R10 and D (p<0.001). Only low levels of a few antibiotics could be detected in the sediments samples (Table S3).

**Table 1:** Average concentrations (R1–3, R5–7, SD n = 3; C1 and C2 n = 2; R4, R8–10, D n = 1) of antibiotics in rivers, dam, canal and vicinity of hospitals (ng L^−1^).

ATB	R1	R2	R3	R4	R5	R6	R7	R8	R9	R10	D	C1	C2	SD
SDZ	<LOQ	46*(70)*	8.0*(140)*	<LOQ	3.2*(140)*	<LOQ	<LOQ	<LOQ	25	<LOQ	<LOQ	20	6.4	<LOQ
LIN	3.6*(100)*	130*(40)*	250*(50)*	0.7	<LOQ	3.5*(60)*	1.9*(40)*	<LOQ	9.2	4.5	1.4	2.0	300	1100*(10)*
TRI	5.8*(140)*	110*(80)*	1700*(110)*	3.2	2.2*(40)*	3.7*(110)*	1.5*(130)*	0.4	11	5.4	4.0	6.1	70	2200 *(60)*
ENX	<LOQ	6.4*(140)*	<LOQ	<LOQ	<LOQ	<LOQ	<LOQ	<LOQ	<LOQ	<LOQ	<LOQ	<LOQ	<LOQ	33*(130)*
OXY	11*(120)*	78*(20)*	1100*(130)*	4.9	3.5*(140)*	6.8*(30)*	4.2*(110)*	1.1	6.4	5.8	<LOQ	14	48	3200*(90)*
OFL	1.1*(140)*	3.5*(100)*	96*(130)*	0.7	0.6*(110)*	1.1*(50)*	<LOQ	<LOQ	0.30*)*	0.4	1.0	0.4	6.0	240*(120)*
LEV	<LOQ	3.2*(90)*	86*(130)*	3.7*)*	0.2*(140)*	<LOQ	<LOQ	<LOQ	<LOQ	<LOQ	<LOQ	<LOQ	6.3	180*(110)*
NOR	2.2*(110)*	<LOQ	38*(130)*	<LOQ	1.4*(80)*	1.9*(50)*	1.0*(70)*	<LOQ	<LOQ	<LOQ	1.6	<LOQ	2.5	4.0*(70)*
PEF	0.9*(100)*	<LOQ	3.8*(110)*	<LOQ	<LOQ	<LOQ	<LOQ	<LOQ	<LOQ	<LOQ	1.1	<LOQ	0.5	3.9*(100)*
CIP	<LOQ	6.1*(100)*	110*(130)*	2.8	0.6*(40)*	1.7*(60)*	<LOQ	<LOQ	<LOQ	<LOQ	1.4	0.6	12	350*(100)*
CEF	<LOQ	<LOQ	16*(130)*	<LOQ	1.7*(80)*	<LOQ	<LOQ	<LOQ	<LOQ	<LOQ	10	3.8	<LOQ	<LOQ
LOM	1.2*(140)*	0.4*(60)*	2.3*(140)*	<LOQ	0.3*(140)*	<LOQ	<LOQ	<LOQ	0.3	<LOQ	0.7	0.4	<LOQ	3.0*(140)*
TET	50*(90)*	31*(60)*	40*(120)*	8.6	12*(60)*	26*(50)*	4.0*(120)*	<LOQ	<LOQ	5.0	6.0	<LOQ	21	14*(60)*
ENR	0.6*(100)*	<LOQ	1.8*(110)*	0.8	<LOQ	<LOQ	<LOQ	<LOQ	<LOQ	<LOQ	<LOQ	<LOQ	<LOQ	0.8*(80)*
AZI	<LOQ	<LOQ	<LOQ	8.6	<LOQ	<LOQ	<LOQ	<LOQ	<LOQ	<LOQ	<LOQ	4.7*)*	21	16*(40)*
CLI	<LOQ	0.6*(70)*	3.1*(140)*	<LOQ	<LOQ	<LOQ	<LOQ	<LOQ	<LOQ	<LOQ	<LOQ	46	6.8	1.0*(20)*
SULM	28*(70)*	890*(80)*	2700*(60)*	67	8.8*(100)*	19*(60)*	15*(140)*	110*)*	32	170	14	42	140	4600*(50)*
DOX	61*(100)*	71*(50)*	300*(120)*	100	13*(40)*	24*(70)*	14*(20)*	9.3	18	11	16	3.6	12	160*(80)*
ERY	11*(90)*	43*(50)*	310*(120)*	3.2	2.4*(30)*	<LOQ	0.7*(20)*	<LOQ	7.6	4.6	7.1	15	66	430*(80)*
NAL	<LOQ	37*(60)*	120*(70)*	13	<LOQ	<LOQ	<LOQ	<LOQ	<LOQ	<LOQ	<LOQ	<LOQ	9.2	190*(120)*
CLA	5.2*(60)*	13*(60)*	37*(120)*	130	1.3*(40)*	1.0*(20)*	0.5*(30)*	2.2	1.1	0.8	1.5	14	50	73*(30)*
ROX	1.0*(80)*	20*(50)*	6.2*(90)*	180	0.9*(70)*	0.7*(80)*	0.9*(50)*	4.2	<LOQ	<LOQ	0.4	3.9	5.8	5.2*(60)*

*ATB:* antibiotics; *R1–R10:* river sites; *D:* dam; *C1–C2:* lahore branch canal; *SD:* sewage drain near hospitals (see Fig. 1 and Table S3); *( )*: RSD (relative standard deviation); *LOQ*: Limit of quantification.

### Determination of antibiotics near pharmaceutical formulation facilities

Results of measured levels of antibiotics in ng L^−1^ near DFFs are presented in Table 2. At sampling site P1a, just before the DFF, the average measured levels were in the range of <LOQ – 70 ng L^−1^ whereas at P1b, downstream the DFF, the levels were in the range <LOQ – 570 ng L^−1^. In both cases the highest concentration measured was of SULM and generally an increasing trend in concentration was seen from P1a to P1b (although not statistically significant) indicating contribution from the DFF. At P2, Sundar Industrial Estate, the range observed was <LOQ – 530 µg L^−1^ with the highest concentration of LIN. At Quaid-e-Azam Industrial Estate, P3, the range observed was <LOQ – 4700 ng L^−1^ with the highest concentration of SULM. The highest levels near a DFF were observed at Shahdara Industrial Estate, P4, ranging from <LOQ – 49000 ng L^−1^ with the highest concentration of SULM. The other antibiotics with the measured levels >1 µg L^−1^ were ERY, LIN, CIP, OFL, LEV, OXY, and TRI with the measured levels of 1100, 4100, 6200, 7300, 8000, 27000 and 28000 ng L^−1^ respectively. With the exception of P1a, the antibiotic levels at the pharmaceutical sampling sites were in general higher than what was measured at the less impacted sites. Sites P1b, P3 and P4 ranked higher in antibiotic levels than R5–R10 and D (p<0.001). Furthermore, P3 had higher antibiotic levels than P1a (p<0.001) and P4 had higher antibiotic levels than P1a and C1 (p<0.001). Only low levels of a few antibiotics could be detected in the sediments samples (Table S3).

**Table 2:** Average concentrations (n = 3) of antibiotics in vicinity of drug formulation facilities (ng L^−1^).

Antibiotics	P1a	P1b	P2	P3	P4
SDZ	19*(80)*	140*(90)*	<LOQ	10*(110)*	290*(30)*
LIN	22*(30)*	270*(40)*	530*(120)*	330*(70)*	4100*(60)*
TRI	18*(50)*	570*(100)*	73*(90)*	1000*(110)*	28000*(100)*
ENX	3.5*(140)*	0.4*(110)*	4.9*(140)*	<LOQ	<LOQ
OXY	<LOQ	160*(50)*	1.2*(120)*	76*(50)*	27000*(90)*
OFL	2.0*(10)*	90*(80)*	6.4*(130)*	120*(60)*	7300*(20)*
LEV	1.8*(10)*	68*(70)*	7.0*(130)*	130*(70)*	8000*(10)*
NOR	<LOQ	<LOQ	4.5*(40)*	45*(60)*	140*(20)*
PEF	<LOQ	1.5*(100)*	<LOQ	8.3*(60)*	82*(20)*
CIP	1.04*(20)*	31*(90)*	2.0*(90)*	91*(70)*	6200*(30)*
CEF	<LOQ	<LOQ	<LOQ	59*(140)*	52*(140)*
LOM	<LOQ	0.8*(100)*	1.0*(110)*	3.4*(40)*	8.7*(30)*
TET	<LOQ	50*(40)*	11*(100)*	28*(70)*	380*(70)*
ENR	<LOQ	7.9*(110)*	0.7*(60)*	3.4*(60)*	5.0*(40)*
AZI	2.0*(30)*	35*(100)*	2.2*(80)*	21*(70)*	8.9*(70)*
CLI	<LOQ	1.0*(20)*	0.6*(40)*	2.2*(110)*	0.2*(70)*
SULM	70*(40)*	520*(50)*	160*(80)*	4700*(100)*	49000*(20)*
DOX	3.0*(10)*	230*(30)*	5.5*(100)*	52*(100)*	370*(20)*
ERY	6.3*(50)*	290*(100)*	34*(60)*	62*(40)*	1100*(10)*
NAL	<LOQ	49*(90)*	16*(60)*	44*(40)*	220*(10)*
CLA	6.2*(20)*	190*(60)*	60*(50)*	360*(30)*	19*(50)*
ROX	1.4*(20)*	240*(60)*	37*(50)*	270*(70)*	3.0*(20)*

*P1a:* upstream Kahuta industrial estate (KIE); *P1b:* downstream KIE; *P2–P4:* drug formulation facilities in and around Lahore (see Fig. 1 and Table S2); *( )*: RSD (relative standard deviation).

### Determination of antibiotic resistance genes and class 1 integrons

**Figure 2:**
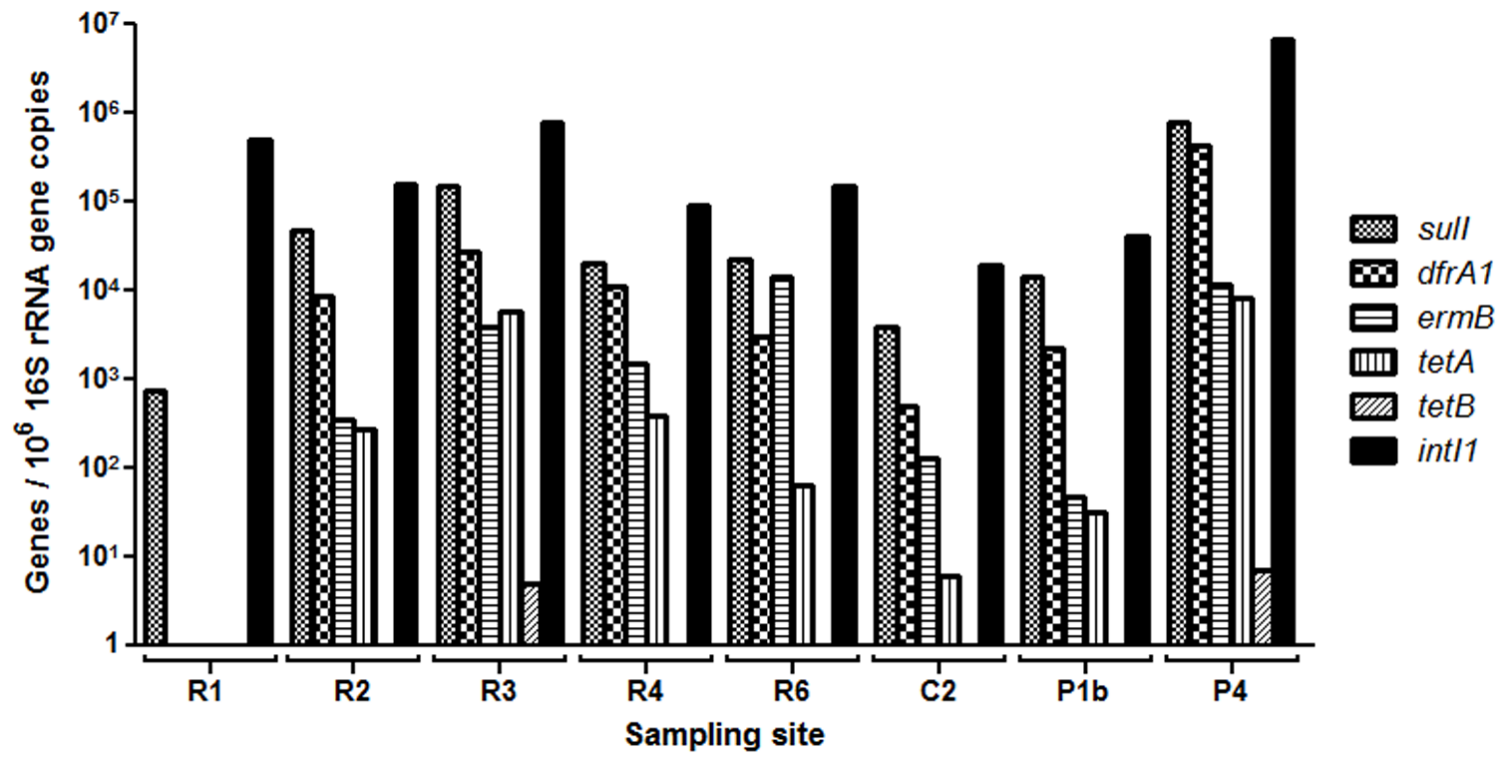
Levels of antibiotic resistance genes (*sulI, dfrA1, erm B, tetA and tetB*) and integrons (*intl1*) at eight sampling sites (n = 1, pooled samples).

Results of measured levels of ARGs and *intl 1* are presented in Table S5 and Figure 2. ARGs could be detected at all river sampling sites but a marked increasing trend was observed from R1 to R3 (p<0.01), i.e. before and after Lahore city. Highest levels were observed at sampling point P4, Shahdara Industrial Estate with levels almost an order of magnitude higher than the river sampling sites. The ARG levels at P4 ranked higher than at R1, C2 and P1b (p<0.05). *sulI* and *dfrA1* in particular were found at high concentrations at P4 (8.0×10^5^ *sulI* copies and 4.3×10^5^ *dfrA1* copies per 10^6^ 16S rRNA gene copies respectively). Class 1 integrons were found in all sediment samples. Site P4 had the highest concentration (6.9×10^6^ *intI1* copies/10^6^ 16S rRNA gene copies) with more than ten times higher concentration than the site with the second highest concentration (R3) and more than 100 times higher than the site with the lowest concentration (R1).

## Discussion

Our measured results of antibiotics in rivers in Northern Pakistan show that most of the investigated rivers are not impacted to a large extent and that the detected levels are comparable to surface water measurements in unpolluted sites in Europe and the US [3]. However, our results also show that large cities, such as Lahore, have a huge impact on the surface water concentration of antibiotics in even major rivers, which results in elevated concentrations of highly biological active compounds in a natural resource used by millions of people.

The integrase gene on class 1 integrons, *intI1*, was also included in the study. Class 1 integrons are one of the most successful elements in the acquisition, maintenance and spread of ARGs and previous reports have showed that sites close to human activities have a higher detection frequency of *intI1* genes [45]–[47]. Interestingly, our results showed that site P4, which had the highest levels of antibiotics measured, also had by far the highest concentration of *intI1*. Furthermore, the measured concentrations of *intI1* increased over 50 times from sampling sites R1 to R3, i.e. upstream and downstream of Lahore. Class 1 integrons have a conserved region that contains *intI1* and the majority also contains *sulI* [46]. Our results show that high levels of ARGs (*sulI* and *dfrA1*) as well as high levels of *intl1* (i.e. class 1 integrons) could indicate a potential correlation to highly antibiotic-contaminated environments. These results imply that there could be a clinical importance of environmental microbial communities since these can act as a reservoir of ARGs and lead to an increase in ARGs in human pathogens.

Studies have revealed that the amount of ARGs in the sediments close to cities is much higher than in pristine environments [48], thus indicating the impact of anthropogenic contamination. When comparing the results of the antibiotic and ARG measurements, both antibiotic and ARG levels were the highest at sampling site P4 and also elevated at R3. Both sites P4 and R3 ranked significantly higher than most sites in overall antibiotic levels. Furthermore, whereas no other site showed any significant differences to any other site in ARG concentrations, P4 and R3 both ranked higher than sites R1, with P4 also ranking higher than sites C2 and P1b. It should be stressed that the ARGs detected at highest levels at sampling site P4, *sulI* and *dfrA1* are directly associated with the antibiotics detected at the highest concentrations, SULM and TRI. This is also true for the sampling point downstream the city of Lahore, R3. However, even though the increase in ARGs at R3 could be explained by a general increase in percentage of waste water at the sampling site, the opposite is true for P4. Sampling site P4 is located at an industrial estate with a low number of local residents and no hospitals, i.e. the waste water at this site is correlated to a low number of people. If the major contributor of ARGs was a clinical source, the levels of ARGs would have to be directly correlated to the number of people connected to a certain volume of waste water. I.e. the level of ARGs would increase if the number of people connected to a specific flow of waste water increased. In the case of sampling point P4, the levels of ARGs are an order of magnitude higher but the number of people connected to the waste water is an order of magnitude lower. This study indicates that the levels of ARGs might reflect the levels of antibiotics in the environment i.e. that a surface water concentration of antibiotics in the range of 1–10 µg L^−1^ can promote antibiotic resistance.

## Supporting Information

Table S1 **Sampling sites coordinates and date(s) of sampling.**
(DOCX)

Table S2 **Selective reaction monitoring transitions used for quantitation and confirmation.**
(DOCX)

Table S3 Average concentrations (n = 3)* of antibiotics in rivers' sediments, canal and nearby drug production facilities (µg Kg^−1^ dw).
(DOCX)

Table S4 **Target genes, primers and cycling conditions for the real-time PCR assays used.**
(DOCX)

Table S5 **Bacterial load and ARG concentrations of sediment of eight sampling sites.** The bacterial load is presented as the number of 16S rDNA copies per mg sediment, and the ARG concentration as gene copies per 10^6^ 16S rDNA copies.
(DOCX)
