# Supplementary material for: Occurrence and Abundance of Antibiotics and Resistance Genes in Rivers, Canal and near Drug Formulation Facilities – A Study in Pakistan
Source: PLoS One. 2013 Jun 28;8(6):e62712. doi: 10.1371/journal.pone.0062712 (PMC3696045; doi:10.1371/journal.pone.0062712)
Supplement: Table S1 — Sampling sites coordinates and date(s) of sampling. (DOCX) [file pone.0062712.s001.docx]

| **Table S1.** |  |  |  |  |
| --- | --- | --- | --- | --- |
| Sampling sites coordinates and date(s) of sampling. | |  |  |  |
| Sample name(s) | Sites | Location | | Sampling date (s) |
|  |  | Latitude | Longitude |  |
| R1 | River Ravi (upstream) | 31.644833 | 74.341135 | 18-01-2012 |
|  |  |  |  | 31-01-2012 |
|  |  |  |  | 15-02-2012 |
| R2 | River Ravi (city) | 31.606537 | 74.296632 | 18-01-2012 |
|  |  |  |  | 31-01-2012 |
|  |  |  |  | 15-02-2012 |
| R3 | River Ravi (downstream) | 31.483832 | 74.156700 | 18-01-2012 |
|  |  |  |  | 31-01-2012 |
|  |  |  |  | 15-02-2012 |
| R4 | River Chenab | 32.481130 | 74.090209 | 04-02-2012 |
| R5 | River Jhelum (upstream) | 32.987392 | 73.763725 | 04-02-2012 |
|  |  |  |  | 11-02-2012 |
|  |  |  |  | 20-02-2012 |
| R6 | River Jhelum (city) | 32.907478 | 73.726673 | 04-02-2012 |
|  |  |  |  | 11-02-2012 |
|  |  |  |  | 20-02-2012 |
| R7 | River Jhelum (downstream) | 32.845955 | 73.657665 | 04-02-2012 |
|  |  |  |  | 11-02-2012 |
|  |  |  |  | 20-02-2012 |
| R8 | River Indus | 33.995750 | 72.423935 | 12-02-2012 |
| R9 | River Kabul | 33.967886 | 72.216461 | 12-02-2012 |
| R10 | River Dor | 34.057388 | 73.149118 | 12-02-2012 |
| D | Rawal Dam | 33.694066 | 73.124142 | 12-02-2012 |
| SD | Sewage Drain (near hospitals) | 31.537661 | 74.331860 | 20-01-2012 |
|  |  |  |  | 31-01-2012 |
|  |  |  |  | 16-02-2012 |
| C1 | Lahore Branch Canal (Jhalomor) | 31.586505 | 74.500662 | 16-02-2012 |
| C2 | Lahore Branch Canal (13 Km Multan Road) | 31.451213 | 74.209127 | 16-02-2012 |
| P1a | Kahuta Industrial Estate (upstream) | 33.544033 | 73.172003 | 04-02-2012 |
|  |  |  |  | 11-02-2012 |
|  |  |  |  | 20-02-2012 |
| P1b | Kahuta Industrial Estate (downstream) | 33.544086 | 73.137027 | 04-02-2012 |
|  |  |  |  | 11-02-2012 |
|  |  |  |  | 20-02-2012 |
| P2 | Sundar Industrial Estate | 31.268516 | 74.177561 | 25-01-2012 |
|  |  |  |  | 07-02-2012 |
|  |  |  |  | 14-02-2012 |
| P3 | Quaid-e-Azam Industrial Estate (Kotlakhpat) | 31.436520 | 74.315099 | 25-01-2012 |
|  |  |  |  | 07-02-2012 |
|  |  |  |  | 14-02-2012 |
