# Supplementary material for: Occurrence and Abundance of Antibiotics and Resistance Genes in Rivers, Canal and near Drug Formulation Facilities – A Study in Pakistan
Source: PLoS One. 2013 Jun 28;8(6):e62712. doi: 10.1371/journal.pone.0062712 (PMC3696045; doi:10.1371/journal.pone.0062712)
Supplement: Table S2 — Selective reaction monitoring transitions used for quantitation and confirmation. (DOCX) [file pone.0062712.s002.docx]

| **Table S2.** | | | | | | | |
| --- | --- | --- | --- | --- | --- | --- | --- |
| Selective reaction monitoring transitions used for quantitation and confirmation. | | | | | | | |
| Antibiotics | *(Class)* | Quantitation | CE | Confirmation | CE | TL | RT |
|  |  | Transitions | (v) | transitions | (v) |  | (min.) |
|  |  | (*m/z*) |  | (*m/z*) |  |  |  |
| SDZ | *(SUL)* | 251.00→92.30 | 26 | 251.00→108.20 | 22 | 77 | 5.20 |
| LIN | *(LIN)* | 407.10→126.20 | 30 | 407.10→359.40 | 18 | 110 | 5.30 |
| TRI | *(DRI)* | 291.00→230.10 | 23 | 291.00→123.20 | 25 | 106 | 5.67 |
| ENX | *(FQN)* | 321.00→302.20 | 19 | 321.00→206.10 | 28 | 110 | 5.71 |
| OXY | *(TET)* | 461.00→426.20 | 18 | 461.00→443.20 | 13 | 130 | 5.81 |
| OFL | *(FQN)* | 362.05→318.20 | 17 | 362.05→261.00 | 25 | 138 | 5.82 |
| LEV | *(FQN)* | 362.00→318.30 | 19 | 362.00→261.20 | 27 | 90 | 5.84 |
| NOR | *(FQN)* | 320.00→302.10 | 15 | 320.00→233.10 | 23 | 114 | 5.84 |
| PEF | *(FQN)* | 334.10→290.20 | 16 | 334.10→233.10 | 24 | 110 | 5.89 |
| CIP | *(FQN)* | 332.00→231.10 | 35 | 332.00→288.20 | 16 | 117 | 5.90 |
| CEF | *(CSN)* | 455.90→396.10 | 5 | 455.90→125.10 | 31 | 106 | 5.92 |
| LOM | *(FQN)* | 352.10→265.20 | 21 | 352.10→308.20 | 15 | 110 | 6.00 |
| TET | *(TET)* | 445.10→410.30 | 18 | 445.10→427.40 | 12 | 109 | 6.04 |
| ENR | *(FQN)* | 360.10→316.00 | 17 | 360.10→245.10 | 25 | 112 | 6.10 |
| AZI | *(MAC)* | 749.40→591.60 | 28 | 749.40→158.20 | 37 | 148 | 6.62 |
| CLI | *(LIN)* | 425.10→126.20 | 31 | 425.10→377.30 | 18 | 110 | 6.88 |
| SULM | *(SUL)* | 254.00→108.20 | 22 | 254.00→156.00 | 15 | 108 | 6.98 |
| DOX | *(TET)* | 445.00→428.30 | 17 | 445.00→200.90 | 27 | 107 | 7.00 |
| ERY | *(MAC)* | 734.30→576.60 | 19 | 734.30→158.10 | 29 | 154 | 7.43 |
| NAL | *(FQN)* | 233.10→215.10 | 14 | 233.10→187.10 | 24 | 85 | 7.89 |
| CLA | *(MAC)* | 748.40→158.10 | 27 | 748.40→590.50 | 17 | 156 | 8.07 |
| ROX | *(MAC)* | 837.40→679.60 | 20 | 837.40→158.10 | 33 | 150 | 8.15 |
| Internal standards | |  |  |  |  |  |  |
| ^13^C_2_-TRI |  | 294.10→233.20 | 22 | 294.10→126.20 | 24 | 101 | 5.68 |
| ^13^C_3_-CIP |  | 336.00→318.00 | 20 | 336.00→291.20 | 18 | 106 | 5.90 |
| ^13^C_6_-SULM | | 260.00→162.10 | 15 | 260.00→98.30 | 25 | 97 | 6.98 |
| ^13^C_2_-ERY | | 736.40→578.60 | 17 | 736.40→160.10 | 29 | 129 | 7.43 |
| *SUL*: sulphonamide; *LIN*: lincosamide; *DRI*: dihydrofolate reductase inhibitor; *FQN*: flouroquinolone; *TET*: tetracycline; *CSN*: cephalosporin; *MAC*: macrolide; CE: collision energy; TL: tube lens; RT: retention time | | | | | | | |
