# Supplementary material for: Occurrence and Abundance of Antibiotics and Resistance Genes in Rivers, Canal and near Drug Formulation Facilities – A Study in Pakistan
Source: PLoS One. 2013 Jun 28;8(6):e62712. doi: 10.1371/journal.pone.0062712 (PMC3696045; doi:10.1371/journal.pone.0062712)
Supplement: Table S3 — Average concentrations (n = 3)* of antibiotics in rivers' sediments, canal and nearby drug production facilities (µg Kg−1 dw). (DOCX) [file pone.0062712.s003.docx]

| **Table S 3 .**  Average concentrations (n=3)* of antibiotics in rivers’ sediments, canal and nearby drug production facilities (µg Kg^-1^ dw). | | | | | | | | |
| --- | --- | --- | --- | --- | --- | --- | --- | --- |
| Antibiotics | R1 | R2 | R3 | R4 | R6 | C2 | P1b | P4 |
| SDZ | <LOQ | <LOQ | <LOQ | <LOQ | <LOQ | <LOQ | <LOQ | <LOQ |
| LIN | <LOQ | <LOQ | <LOQ | <LOQ | <LOQ | <LOQ | <LOQ | <LOQ |
| TRI | <LOQ | 2.2*(0.5)* | 80*(2.0)* | <LOQ | <LOQ | <LOQ | 2.8*(16)* | 2.5*(8.4)* |
| ENX | <LOQ | <LOQ | <LOQ | <LOQ | <LOQ | <LOQ | <LOQ | <LOQ |
| OXY | 26*(8.0)* | 22*(4.0)* | 19*(4.0)* | <LOQ | 11*(1.0)* | 50*(6.0)* | 17*(4.0)* | 24*(9.0)* |
| OFL | 5.7*(0.2)* | 10*(1.0)* | 74*(3.0)* | <LOQ | <LOQ | 3.2*(0.4)* | <LOQ | 2.7*(0.1)* |
| LEV | 5.6*(1.0)* | 11*(1.0)* | 78*(3.0)* | <LOQ | <LOQ | <LOQ | <LOQ | 2.3*(2.0)* |
| NOR | <LOQ | <LOQ | <LOQ | <LOQ | <LOQ | <LOQ | <LOQ | <LOQ |
| PEF | <LOQ | <LOQ | <LOQ | <LOQ | <LOQ | <LOQ | <LOQ | <LOQ |
| CIP | <LOQ | 17*(1.0)* | 23*(6.0)* | <LOQ | <LOQ | 23*(0.2)* | <LOQ | <LOQ |
| CEF | <LOQ | <LOQ | <LOQ | <LOQ | <LOQ | <LOQ | <LOQ | <LOQ |
| LOM | <LOQ | <LOQ | <LOQ | <LOQ | <LOQ | <LOQ | <LOQ | <LOQ |
| TET | <LOQ | <LOQ | <LOQ | <LOQ | <LOQ | <LOQ | <LOQ | <LOQ |
| MEC | <LOQ | <LOQ | <LOQ | <LOQ | <LOQ | <LOQ | <LOQ | <LOQ |
| ENR | <LOQ | <LOQ | <LOQ | <LOQ | <LOQ | <LOQ | <LOQ | <LOQ |
| AZI | <LOQ | <LOQ | <LOQ | <LOQ | <LOQ | <LOQ | <LOQ | <LOQ |
| CLI | <LOQ | <LOQ | <LOQ | <LOQ | <LOQ | <LOQ | <LOQ | <LOQ |
| SULM | <LOQ | <LOQ | 6.1*(0.2)* | <LOQ | <LOQ | 4.1*(0.1)* | 4.1*(0.1)* | <LOQ |
| DOX | <LOQ | <LOQ | <LOQ | <LOQ | <LOQ | <LOQ | <LOQ | <LOQ |
| ERY | <LOQ | <LOQ | <LOQ | <LOQ | 3.0*(0.3)* | <LOQ | <LOQ | <LOQ |
| NAL | 43*(15)* | 30*(6.0)* | 8.6*(20)* | 2.1*(5.0)* | <LOQ | <LOQ | <LOQ | 5.2*(6.0)* |
| CLA | <LOQ | <LOQ | <LOQ | <LOQ | <LOQ | <LOQ | <LOQ | <LOQ |
| ROX | <LOQ | <LOQ | <LOQ | <LOQ | <LOQ | <LOQ | <LOQ | <LOQ |
| CLO | <LOQ | <LOQ | <LOQ | <LOQ | <LOQ | <LOQ | <LOQ | <LOQ |
| *R1-R3:* River Ravi (upstream, city and downstream); *R4:* River Chenab; *R6:* River Jhelum (city); *C2:* Lahore branch canal (downstream); *P1b:* Kahuta industrial estate (downstream); *P4:* Shahdara industrial estate(see Fig.1); *dw:* dry weight; *( )* RSD (relative standard deviation); *: three analysis of the same sample. | | | | | | | | |
