# Supplementary material for: Occurrence and Abundance of Antibiotics and Resistance Genes in Rivers, Canal and near Drug Formulation Facilities – A Study in Pakistan
Source: PLoS One. 2013 Jun 28;8(6):e62712. doi: 10.1371/journal.pone.0062712 (PMC3696045; doi:10.1371/journal.pone.0062712)
Supplement: Table S4 — Target genes, primers and cycling conditions for the real-time PCR assays used. (DOCX) [file pone.0062712.s004.docx]

| **Table S4** |  |  |  |  |  |  |  |  |  |  |
| --- | --- | --- | --- | --- | --- | --- | --- | --- | --- | --- |
| Target genes, primers and cycling conditions for the real-time PCR assays used. | | | | | | | | | | |
| **Primer** | **Gene** | **Sequence (5' to 3')** | **Primer** | **Annealing** | **Cycles** | | **Detection format** | | **Reference** | |
|  |  |  | **amount (pmol)** | **temp. (°C)** |  |  |  |  |  |  |
| 341F | 16S rDNA | CCT ACG GGA GGC AGC AG | 6.0 | 60 | 30 | | SYBR Green | | *49* | |
| 534R |  | ATT ACC GCG GCT GCT GGC A |  |  |  |  |  |  |  |  |
| tetA-F2-L | *tetA* | CAG CCT CAA TTT CCT GAC GGG CtG * | 4.0 | 60 | 45 | | LUX | | *50* | |
| tetA-R2 |  | GAA GCG AGC GGG TTG AGA G |  |  |  |  |  |  |  |  |
| tetB-F1-L | *tetB* | CAG CAA GTG CGC TTT GGA TGC tG * | 4.0 | 60 | 45 | | LUX | | *50* | |
| tetB-R1 |  | TGA GGT GGT ATC GGC AAT GA |  |  |  |  |  |  |  |  |
| erm(B)-91f | *ermB* | GAT ACC GTT TAC GAA ATT GG | 5.0 | 58 ‡ | 45 ‡ | | SYBR Green | | *51* | |
| erm(B)-454r |  | GAA TCG AGA CTT GAG TGT GC |  |  |  |  |  |  |  |  |
| sulI-FW | *sulI* | CGC ACC GGA AAC ATC GCT GCA C | 4.0 | 65 | 50 | | SYBR Green | | *48* | |
| sulI-RV |  | TGA AGT TCC GCC GCA AGG CTC G |  |  |  |  |  |  |  |  |
| dfr1s-f | *dfr1* | ATG GAG TGC CAA AGG TGA AC | 10 | 62 | 40 | | SYBR Green | | *52* | |
| dfr1s-r |  | TAT CTC CCC ACC ACC TGA AA |  |  |  |  |  |  |  |  |
| int1.F | *intI1* | GGG TCA AGG ATC TGG ATT TCG | 10 | 55 | 45 | | SYBR Green | | *53* | |
| int1.R |  | ACA TGC GTG TAA ATC ATC GTC G |  |  |  |  |  |  |  |  |
| * Lower-case letter 't' denotes a thymine base with a FAM dye attached. | | | | | | | | | | |
| † Probe is modified with a FAM dye at the 5'-end and a Black Hole Quencher at the 3'-end. | | | | | | | | | | |
| ‡ Initial touchdown step of 63 °C – 1 °C / cycle, for 5 cycles | | | | | | | | | | |

**References**

49. Bru D, Martin-Laurent F, Philippot L (2008) Quantification of the detrimental effect of a single primer-template mismatch be real-time PCR using the 16S rRNA gene as an example. Appl Environ Microbiol 74: 1660-1663.

50. Börjesson S, Mattsson A, Lindgren PE (2010) Genes encoding tetracycline resistance in a full-scale municipal wastewater treatment plant investigated during one year. J Water Health 8(2): 247-256.

51 Chen J, Yu ZT, Michel FC, Wittum T, Morrison M (2007) Development and application of real-time PCR assays for quantification of erm genes conferring resistance to macrolides-lincosamides-streptogramin B in livestock manure and manure management systems. Appl Environ Microbiol 73(14): 4407-4416.

52 Grape M, Motakefi A, Pavuluri S, Kahlmeter G (2007) Standard and real-time multiplex PCR methods for detection of trimethoprim resistance dfr genes in large collections of bacteria. Clin Microbiol Infect 13(11): 1112-1118.

53. Mazel D, Dychinco B, Webb VA, Davies J (2000) Antibiotic resistance in the ECOR collection: integrons and identification of a novel aad gene. Antimicrob Agents Chemother 44(6): 1568-1574.
