# Supplementary material for: Occurrence and Abundance of Antibiotics and Resistance Genes in Rivers, Canal and near Drug Formulation Facilities – A Study in Pakistan
Source: PLoS One. 2013 Jun 28;8(6):e62712. doi: 10.1371/journal.pone.0062712 (PMC3696045; doi:10.1371/journal.pone.0062712)
Supplement: Table S5 — Bacterial load and ARG concentrations of sediment of eight sampling sites. The bacterial load is presented as the number of 16S rDNA copies per mg sediment, and the ARG concentration as gene copies per 106 16S rDNA copies. (DOCX) [file pone.0062712.s005.docx]

**Table S5.**

Bacterial load and ARG concentrations of sediment of eight sampling sites. The bacterial load is presented as the number of 16S rDNA copies per mg sediment, and the ARG concentration as gene copies per 10^6^ 16S rDNA copies.

| Sample site | 16S rDNA (mg^-1^) | Gene copies per 10^6^ 16S rDNA copies | | | | | |
| --- | --- | --- | --- | --- | --- | --- | --- |
|  |  | *sulI* | *dfr1* | *ermB* | *tetA* | *tetB* | *intI1* |
| R1 | 240000 | 730 | <LOQ | <LOQ | <LOQ | <LOQ | 500000 |
| R2 | 3800000 | 48000 | 8600 | 360 | 270 | <LOQ | 160000 |
| R3 | 3000000 | 150000 | 28000 | 3800 | 5700 | 5 | 770000 |
| R4 | 1000000 | 20000 | 11000 | 1500 | 390 | <LOQ | 93000 |
| R6 | 4500000 | 22000 | 3100 | 14000 | 63 | <LOQ | 150000 |
| C2 | 5000000 | 3800 | 490 | 130 | 6 | <LOQ | 19000 |
| P1b | 4000000 | 14000 | 2200 | 47 | 32 | <LOQ | 41000 |
| P4 | 1100000 | 800000 | 430000 | 12000 | 8400 | 7 | 6900000 |
